# Emerging Implications for Extracellular Matrix-Based Technologies in Vascularized Composite Allotransplantation

**DOI:** 10.1155/2016/1541823

**Published:** 2015-12-29

**Authors:** Ricardo Londono, Vijay S. Gorantla, Stephen F. Badylak

**Affiliations:** ^1^McGowan Institute for Regenerative Medicine, University of Pittsburgh, Pittsburgh, PA 152192, USA; ^2^School of Medicine, University of Pittsburgh, Pittsburgh, PA 152613, USA; ^3^Department of Bioengineering, University of Pittsburgh, Pittsburgh, PA 152134, USA; ^4^Department of Plastic Surgery, University of Pittsburgh Medical Center, Pittsburgh, PA 152615, USA; ^5^Department of Surgery, University of Pittsburgh, Pittsburgh, PA 15261, USA

## Abstract

Despite recent progress in vascularized composite allotransplantation (VCA), limitations including complex, high dose immunosuppression regimens, lifelong risk of toxicity from immunosuppressants, acute and most critically chronic graft rejection, and suboptimal nerve regeneration remain particularly challenging obstacles restricting clinical progress. When properly configured, customized, and implemented, biomaterials derived from the extracellular matrix (ECM) retain bioactive molecules and immunomodulatory properties that can promote stem cell migration, proliferation and differentiation, and constructive functional tissue remodeling. The present paper reviews the emerging implications of ECM-based technologies in VCA, including local immunomodulation, tissue repair, nerve regeneration, minimally invasive graft targeted drug delivery, stem cell transplantation, and other donor graft manipulation.

## 1. Vascularized Composite Allotransplantation

The field of reconstructive surgery has made significant progress in the last few decades. Despite advancements in technique and instrumentation, severe trauma and/or congenital abnormalities necessitating complicated and extensive tissue reconstruction remain challenging clinical problems. Well established strategies to address these conditions include the use of complex techniques such as bone and muscle grafts, partial/full thickness dermal flaps, and composite tissue flaps. Nevertheless, these techniques are hampered by nontrivial complications such as morbidity at the donor site, limited availability of autologous tissues, and complications associated with extensive surgery [1–3]. Such problems are compounded by the high costs associated with multiple surgical procedures, extended hospital stay, and strenuous rehabilitation.

Novel strategies to circumvent these issues have recently emerged. Vascularized composite allotransplantation (VCA) is a promising field that investigates the transplantation of composite anatomic homologous structures from immunologically and aesthetically compatible donors. Using this approach, close to 200 VCA procedures have been successfully performed worldwide in the last decade, including more than 110 hand transplants and 35 facial transplants [4]. Overall, VCA has achieved encouraging graft survival rates and functional outcomes. With few exceptions, patients who have complied with their treatment regimens have experienced satisfactory restoration of significant tissue deficits, improved functional and aesthetic outcomes, and reduced complications associated with these procedures [5–7].

**Figure 1:**
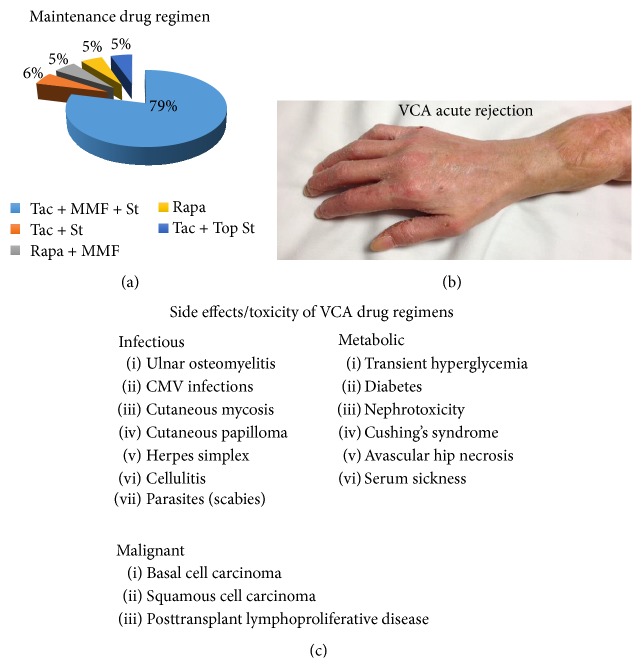
Limitations associated with VCA: Limitations and complications currently associated with VCA include (a) the lifelong need for high dose, multidrug immunosuppressive medications, (b) acute (and chronic) VCA rejection, and (c) side effects and toxicity of antirejection therapies. Patient noncompliance is the main cause of graft loss. Tac: tacrolimus, MMF: Mycophenolate mofetil, Rapa: Rapamycin, St: Steroid, and Top: topical.

Despite promising results enabling independence with activities of daily living and social or professional reintegration of patients, a number of important obstacles and limitations persist with VCA. These limitations include (1) multidrug immunosuppression regimens [8, 9], (2) serious systemic side effects and toxicity from immunosuppressants including the risk of life threatening, life shortening, or quality of life reducing complications [6, 10], (3) acute and chronic allograft rejection [6], and (4) suboptimal nerve regeneration negatively impacting overall motor or sensory functional outcomes [11–14] (Figure 1 and Table 1).

## 2. Novel Strategies and Implications in VCA 

### 2.1. Drug Delivery Applications

**Figure 2:**
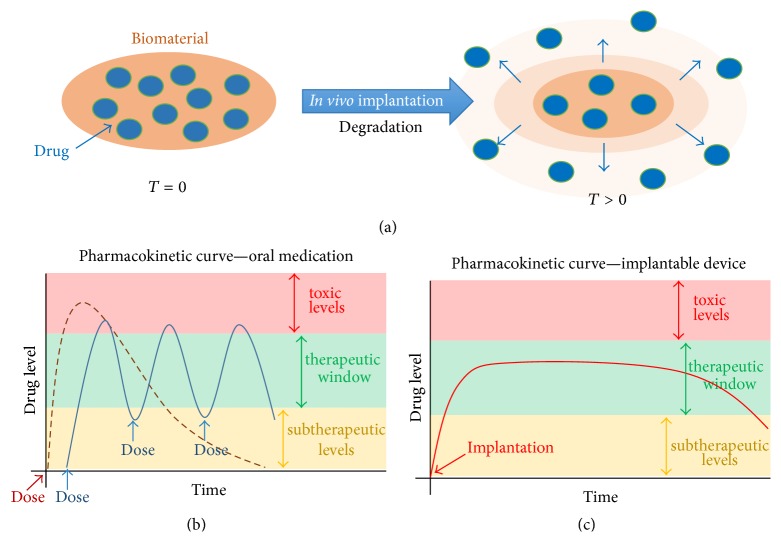
Implantable devices for drug localized delivery. Minimally invasive implantation of drug eluting biomaterials offers advantages over oral drug regimens including localized drug delivery, minimizing or eliminating systemic side effects, and overcoming the barrier of patient noncompliance. (a) Graft implanted biomaterials release active drugs or prodrugs in controlled fashion upon* in vivo* degradation directly into the local tissue microenvironment. (b) Pharmacokinetic profiles of oral medication versus. (c) Implantable drug eluting materials. Additional advantages include reduced dosing, frequency and duration of dosing, favorable drug kinetics and maintenance of pharmacologic agents within the therapeutic window limiting systemic exposure.

A number of technologies are currently under investigation to address the limitations associated with VCA. Local delivery of immunosuppressive agents directly into the graft is a promising alternative to oral medication intake [15]. VCA, unlike other solid organs, are accessible for targeted interventional therapies and visible for clinical and histologic monitoring of grafts. Most systemic immunosuppressive agents are associated with high toxicity and/or narrow therapeutic windows of efficacy. Hence, the main purpose of graft targeted drug delivery strategies is to mitigate systemic exposure and adverse drug related side effects by localizing the delivery of therapeutic agents to the treatment site. Additional advantages of such an approach would include the minimization of overall dosing, further reducing complications associated side effects and toxicity, minimization of frequency of dosing, further reducing complications associated with patient noncompliance, and ease of removal, should an allergic or adverse reaction be experienced by the patient (Figure 2).

The concept of drug eluting biomaterials as implantable delivery systems is not novel. Originally conceived in the 1930s by Deanesly and Parks, this concept was further expanded in the 1960s by Folkman and Long with the investigation of implantable formulations with drug release rates controlled by a rate controlling membrane [16, 17]. Significant progress has been made since implantable drug delivery systems were first conceived. However, commercially available technologies for clinical use are still limited to drug eluting stents for cardiovascular applications [18, 19], intrauterine devices for contraception and treatment of diseases [20, 21], and intraocular inserts for the treatment of glaucoma and cytomegalovirus retinitis [22].

Technologies under development include a broader spectrum of biomaterials and applications. Latest generation technologies such as smart materials that can respond to environmental cues including temperature-responsive [23–27], pH-responsive [28–32], and solvent-responsive [33, 34] polymer-based drug delivery systems [35] offer greater control over their pharmacokinetic properties and drug release profiles. Highly complex bioresponsive materials include hydrogel-based on-demand drug delivery systems such as polysaccharide-based hydrogels that can release matrix metalloprotease (MMP) inhibitors in response to MMP activity [36], and reloadable constructs designed to circumvent serial implantations upon active ingredient depletion [37].

Despite promising results, limitations associated with implantable drug delivery systems that are particularly relevant to VCA remain to be addressed. The foreign body reaction and proinflammatory microenvironment associated with synthetically derived biomaterials [38, 39], as well as the residual effects associated with these technologies, particularly following consecutive implantations, are among the most important limitations.

### 2.2. Stem Cell-Based Therapies

Cell-based attempts to modulate the inflammatory response thereby enhancing graft survival following allotransplantation rely heavily upon the privileged immunological properties of stem cells. The objective of this approach is to harness the immunomodulatory properties of stem cells to induce long term tolerance and graft survival by delivering stem cells into the host.

The immunomodulatory properties of stem cells have been attributed to a low immunostimulatory profile as well as to active immunomodulatory processes both locally as well as a paracrine phenomenon. The low immunostimulatory profile is attributed to decreased antigenicity due to low levels of MHC-I molecule expression and lack of MHC-II expression in both embryonic stem cells (ESC) [40–45] and induced pluripotent stem cells (iPSC) [46–49]. On the other hand, active immunomodulation [42, 44, 50–52] has been shown to be the result of both ligand-mediated [42, 50] and soluble factor-mediated [53, 54] processes. Of these factors, prostaglandin-E2 (PGE2) has been identified as a potent soluble mediator of ESC-mediated immune suppression [55].

Initial* in vivo* studies reported partial success of ESC engraftment and avoidance of immune system activation. However, these experiments were carried out in immunodeficient animals [44, 53, 56, 57], and further work performed in wild type animals in more recent studies contradicted these initial results [58–60]. Although ESCs are still not considered a definite and reliable therapeutic agent for long term graft tolerance induction [61], bone marrow-derived and adipose-derived mesenchymal stem cells (MSCs) have recently emerged as potential immunomodulatory agents [62, 63] that have been shown to promote hematopoietic stem cell engraftment [64, 65] in solid organ transplantation [66, 67] and graft-versus-host disease [68] and to prolong graft survival in a preclinical model of VCA [69].

### 2.3. Tolerance Approaches

In contrast to other therapies, the advantage of establishing immune tolerance is to ideally prevent/control both acute and chronic rejection without maintenance therapy therefore reducing the overall burden of immunosuppression [9].

Although the mechanisms of immune tolerance are not entirely understood, spontaneous tolerance has been reported in liver and kidney transplants [70, 71], and associated with transient chimerism in renal transplant patients [72–74]. However, there appears to be an immunogenic hierarchy among different organs. While a significant number of liver transplant patients may spontaneously develop tolerance, and transient chimerism might be sufficient for tolerance development in renal transplant patients, VCA have reported to be particularly immunogenic and as a result, the establishment of mixed, stable chimerism seems to be the most promising option [75, 76]. In fact, while simultaneous transplantation of stem cells and VCA has been shown to achieve tolerance in experimental studies [9, 69], infusion of donor bone marrow has been shown to be immunomodulatory and potential facilitator for the eventual transition to maintenance immunosuppression with single-agent tacrolimus [48].

In summary, although adjuvant therapies under development are promising and address some of the issues associated with VCA, several nontrivial obstacles remain to be addressed including the well-described proinflammatory host response upon implantation of synthetic materials used in drug and cell delivery, immune system modulation, suboptimal nerve regeneration, and the necessity of at least partial systemic immunosuppression.

## 3. Extracellular Matrix-Derived Biomaterials and Reconstructive Surgery

### 3.1. The Extracellular Matrix

The ECM is a complex milieu of both structural and functional molecules that is secreted by the resident cell population of every tissue and organ. Every tissue is unique in its exact composition and 3-dimensional ultrastructural organization of the ECM. Collagen type I is the main component of the ECM in most tissues, comprising more than 90% of its mass [77, 78]. Other molecules such as laminin, fibronectin, glycosaminoclycans, and other types of collagen are also present in the ECM in various proportions depending upon the specific tissue type to which they belong. For example, while tissues with a basement membrane have a higher proportion of collagen type IV [79], tendons and ligaments need a higher proportion of type I collagen to withstand the mechanical loads to which they are subjected [80]. Vascular tissues need to be flexible and elastic and hence have a higher proportion of laminin and elastin [81]. The ECM is responsible, at least in part, for the diverse mechanical properties found across tissues and organs, and therefore, its composition reflects the mechanical and physiologic demands of every tissue [39].

In addition to providing structural support, the ECM provides biochemical and mechanical cues to the cells of every tissue. The ECM participates in signal transduction directly by interacting with cell receptors, and/or indirectly by facilitating connectivity between adjacent cells [82, 83]. The ECM binds, sequesters, and stores signaling molecules that are released or exposed when needed. Among the diverse physiologic properties influenced by the ECM are stem/progenitor cell migration and chemotaxis, and modulation of the innate and acquired immune systems. In addition to serving as a reservoir for signaling molecules [84–86], the ECM can also communicate physical stimuli to local cell populations by being physically attached to cell receptors that transduce these cues to the cytoskeleton [87]. Hence, the ECM exists in a state of dynamic reciprocity with the microenvironment. While local cell populations secrete the ECM, the ECM itself can in turn influence cell signaling and behavior and modify gene expression profiles [88]. For this reason, the ECM is an important mediator of homeostasis both in health and disease.

### 3.2. Extracellular Matrix-Derived Biomaterials

**Figure 3:**
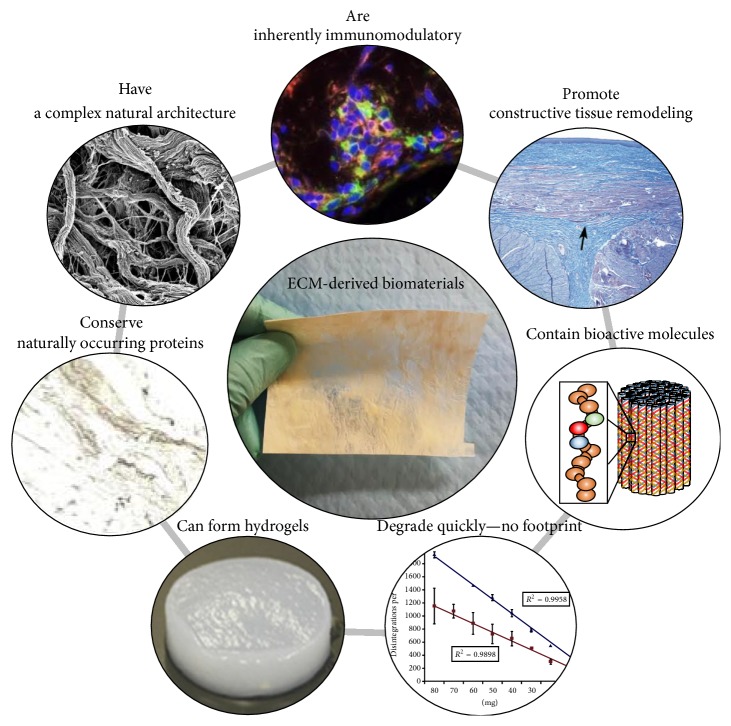
Extracellular matrix-derived biomaterials: Materials derived from the ECM via decellularization of native tissues possess a number of beneficial properties that together promote constructive tissue remodeling. ECM-derived biomaterials can modulate wound healing mechanisms including angiogenesis and the immune system and facilitate important cellular processes such as cell migration, proliferation, and differentiation.

ECM-derived biomaterials are typically manufactured by means of tissue decellularization. Although the exact composition and structure of the ECM varies from one tissue source to another, the main components of the ECM are generally conserved across different tissues and even across different species. This principle is the basis for the successful clinical implementation of regenerative medicine strategies using biomaterials originating from xenogeneic sources (Figure 3).

The objective of the decellularization process is to remove immunogenic and proinflammatory material (i.e., cellular components) that could elicit an adverse immune response [89, 90] while conserving the structure and composition of the ECM, both of which have beneficial effect in the tissue repair process. Specific details concerning the decellularization process have been reviewed elsewhere [91].

To date, a variety of source tissues have been successfully decellularized and products composed of mammalian ECM are commercially available and routinely used in surgical applications (Table 2). ECM-derived biomaterials have been used clinically in multiple reconstructive surgical applications including breast reconstruction [92], ventral hernia repair [93], facial reconstruction [94], and skeletal muscle repair [95], among others (Table 3). The clinical success of these therapies depends on a number of factors including both host-related factors and biomaterial-related factors. Host-related factors include age, comorbidities such as diabetes and obesity, and immunocompetence status, among others. The ECM-related factors include the composition and ultrastructure of the biomaterial, degradability, mechanical properties such as elasticity and compliance, and surface topography. The species and tissues from which the ECM material is procured [96], the decellularization and manufacturing process [91], and postprocessing modifications such as cross-linking [97], solubilization [98–101], and terminal sterilization [39, 102] are also important factors in clinical outcome. As a result, the clinical success of an ECM bioscaffold approach varies widely.

Studies have shown that constructive tissue remodeling, a term that implies the synthesis of site-appropriate, functional tissue, consistently occurs when (1) the materials are appropriately decellularized [89, 90], (2) chemical cross-linking is avoided [39, 97], (3) the materials are free of endotoxin and bacterial contamination [110], (4) the material is placed in contact with healthy and vascularized surrounding tissue [96], and (5) the implant site is subjected to appropriate physiologic and mechanical loads and stimuli [95, 111].

### 3.3. Biomaterial-Mediated Tissue Repair

**Figure 4:**
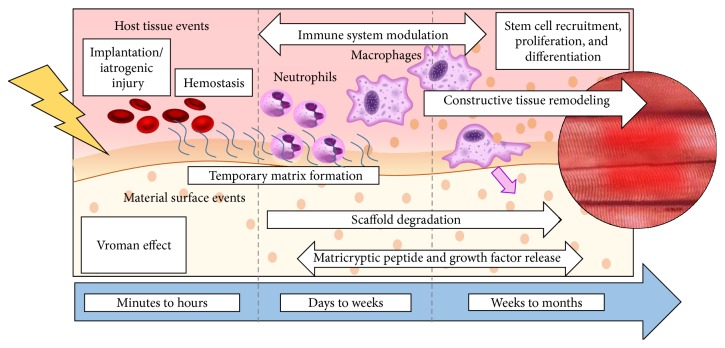
Biomaterial-mediated tissue repair: The biomaterial-host interaction is a complex process composed of multiples stages. Following implantation, the surface of the material is covered with blood and plasma protein through a process known as the Vroman effect. In conjunction with hemostasis, the Vroman effect facilitates the formation of a temporary fibrin-rich matrix that mediates the interaction between native tissues and the implanted construct. This temporary matrix also facilitates cellular access into the material. The innate immune system is activated and a neutrophil accumulation at the periphery of the implant becomes histologically apparent within minutes to hours of implantation. In the following days, this neutrophil accumulation is gradually replaced by a macrophage infiltrate that facilitates scaffold degradation and matricryptic peptide release. The macrophage infiltrate then transitions from an M1 proinflammatory phenotype into an M2 proremodeling phenotype. Signaling molecules produced by the innate immune system and scaffold degradation products act synergistically to recruit stem/progenitor cells from nearby tissues and the bone marrow. Together this heterogeneous cell population known as the constructive cell infiltrate is responsible for further scaffold degradation, neomatrix deposition, and constructive functional tissue remodeling.

ECM biomaterials have the ability to change the default postnatal wound healing response from inflammation and scar tissue formation to site-appropriate constructive tissue remodeling (Figure 4). The properties that enable ECM-derived biomaterials to facilitate such a dramatic switch are inherent to the composition and structure of the ECM itself. The ECM has a complex structure and composition that are created by the local cell population of every tissue, and hence, it is thought to be an ideal and highly biocompatible substrate for regenerative medicine applications.

The host response to implanted biomaterials begins with activation of the innate immune system. Within minutes to hours, the implanted construct adsorbs blood and plasma proteins to its surface through a phenomenon known as the Vroman effect [112]. In combination with hemostasis and clot formation, the Vroman effect leads to the formation of a temporary fibrin-rich matrix that surrounds the implanted construct. This temporary matrix serves to facilitate cellular access to the material. Within hours of implantation, a neutrophil infiltrate accumulates at the surface interface of the implant. Neutrophils play important roles in the host biomaterial interaction including secretion of proteolytic enzymes and secretion of cytokines and chemokines which initiate and modulate subsequent phases of the host response. The neutrophil response is rapidly replaced by a macrophage infiltrate that becomes dominant as early as 2-3 days after implantation and can last for several months. Macrophages secrete additional proteolytic enzymes that degrade the implanted ECM construct, release bioactive cryptic peptides from the parent ECM molecules, and facilitate the deposition of new host-derived tissues.

Signaling molecules released from biologic scaffolds promote a phenotypic change in the macrophage infiltrate from an M1 proinflammatory phenotype to an M2 immunomodulatory phenotype. Biologic materials that can promote an M2 immunomodulatory macrophage phenotype are consistently associated with downstream constructive remodeling events, whereas biologic materials that promote an M1 proinflammatory phenotype are consistently associated with less favorable clinical outcomes including scar formation, encapsulation, foreign body reaction, and, in some cases, seroma formation.

The rate of scaffold degradation depends on a number of factors including the tissue source from which they are derived, and the manufacturing process. For example, while the dense structure of dermal ECM scaffolds prolongs* in vivo* degradation, ECM scaffolds derived from less dense tissues such as the small intestinal submucosa or the urinary bladder are rapidly degraded* in vivo* [113, 114]. Inhibition of ECM scaffold degradation by either chemically crosslinking methods or* in vivo* macrophage depletion prevents the release or exposure of matricryptic peptides and bioactive molecules from the parent molecules such as collagen and laminin within the ECM.

Matricryptic peptides are molecular subunits of larger parent molecules that have biologic activity and can influence cell behavior. Many such oligopeptides have been identified and a more extensive description can be found elsewhere [115, 116]. These cryptic peptides can affect cell migration, proliferation, and differentiation, all of which are important processes for wound healing and tissue repair, and which can provide key components for functional tissue reconstruction in plastic and reconstructive surgery. Matricryptic peptides are thought to have evolved as a source of signals for tissue repair following natural ECM degradation following tissue injury.

## 4. Emerging ECM-Based Technologies 

As stated previously, some of the major obstacles encountered in the field of VCA include high immunogenicity of transplanted tissues, patient noncompliance with increasingly complex immunosuppressive regimens, severe side effects from systemic immunosuppression, and suboptimal nerve regeneration. ECM-derived technologies that are currently under development are potential strategies to address these issues.

### 4.1. ECM-Based Drug Delivery Technologies

A proposed alternative for reducing the amount of oral immunosuppressive agents taken by patients and hence reducing noncompliance and systemic side effects and toxicity is the development of implantable drug eluting biomaterials. However, as previously discussed, most existing drug eluting biomaterials are synthetically derived, and such biomaterials are typically associated with an aggressive inflammatory response and/or foreign body reaction [38, 39]. Seroma, scar tissue formation, and fibrotic encapsulation are all processes that have been associated with implantable synthetic biomaterials. These unfavorable outcomes may not only interfere with release rates and pharmacokinetic profiles of drug eluting materials by creating a physical barrier between the implant and target host tissues, but can also promote a proinflammatory state that can trigger graft rejection.

**Figure 5:**
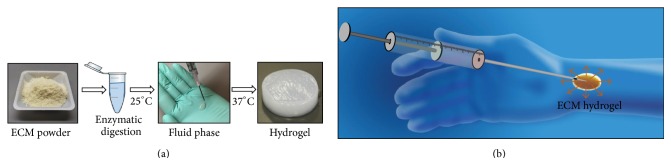
Minimally invasive delivery of ECM-based hydrogels. (a) ECM-derived hydrogels can be manufactured via enzymatic digestion of powdered ECM in an acidic 1 mg/mL pepsin solution. Gelation is induced by neutralizing the pH and salt concentration with 0.1 N NaOH solution followed by warming to 37°C. (b) ECM-derived hydrogels exist in a liquid phase at 25°C and therefore can be delivered minimally invasively via injection. Once* in situ*, hydrogels reach thermal equilibrium with the surrounding tissue and polymerize spontaneously at 37°C. Their degradation profile and biocompatible properties make ECM-derived hydrogels potential candidates for drug delivery applications.

Injectable hydrogels derived from synthetic polymers with tunable structural, chemical, and mechanical properties have traditionally been investigated for drug delivery applications [23, 117, 118]. Similarly, injectable hydrogels derived from naturally occurring biologic materials with superior biocompatibility and bioactivity compared to their synthetic counterparts are currently under development and their immunomodulatory properties are particularly relevant for VCA applications [98–100, 119–122]. The most common components of biologic hydrogels include type I collagen, hyaluronic acid, and other structural proteins such as laminin [122]. ECM-derived hydrogels provide an environment of naturally occurring molecules that allow for cellular infiltration and neovascularization in ischemic regions [123]. ECM-derived hydrogels exist as solutions or suspensions at room temperature 25°C and self-assemble to a gel state at body temperature 37°C [122, 124]. These favorable properties permit ECM-derived hydrogels to be manufactured and stored in liquid phase for minimally invasive deployment via injection and to self-assemble* in situ*, once they equilibrate with body temperature at 37°C (Figure 5). Furthermore, ECM hydrogels retain the properties of traditional ECM-derived biomaterials and therefore are completely degradable with no residual collateral tissue sequelae, are immunomodulatory, and can be replaced by site-appropriate, functional tissue.

### 4.2. ECM-Based Technologies for Cell Delivery

The potential of stem cell transplantation for immunomodulation as adjuvant treatment to immunosuppression has been demonstrated in solid organ transplantation and in preclinical models of VCA [69, 125]. Despite uncontrolled differentiation, low survival, and minimal integration upon implantation, stem cells have been shown to home to sites of injury and inflammation [126, 127], and transplanted tissue [125] and to exert their immunomodulatory effects via paracrine mechanisms [128, 129]. Cell transplantation directly into tissue deficits constitutes a viable alternative for the use of stem cells in VCA. However, the key to cell survival is their ability to integrate with adjacent tissues, and the ideal vehicle for stem cell delivery remains to be determined.

Biomaterials that promote improved cell survival could serve as delivery vehicles for VCA applications. Deployment of stem cells through minimally invasive hydrogel-based technologies offers many advantages such as minimization of iatrogenic injury during implantation, and the presence of a supportive microenvironment to promote cell engraftment. For example, a hydrogel blend of hyaluronan and methylcellulose has been shown to improve cell survival and integration of retinal stem cell-derived rods in the retina, as well as the distribution, viability, and functional repair of neural stem and progenitor cells [130]. Engineered hydrogel matrices have also been shown to provide biomimetic environments to promote cell growth and differentiation [131], a property that is perhaps more prominent in hydrogels derived directly from the ECM because these constructs retain the inherent bioactivity of the native matrix and can promote tissue repair. Furthermore, the immunomodulatory properties of ECM-derived materials could act synergistically with the immunomodulatory properties of stem cells [98, 99, 118, 119, 122, 132] and create an ideal anti-inflammatory microenvironment for tissue repair and allograft survival.

In short, ECM-derived hydrogels provide important biologic support to important cell and clinically relevant functions. Minimally invasive delivery methods combined with the immunomodulatory properties of these materials suggest a promising alternative for the delivery of stem cell populations as adjuvant immunomodulatory therapy in the setting of VCA.

### 4.3. ECM-Induced Nerve Regeneration

Nerve regeneration within transplanted allografts is currently one of the main challenges in VCA [11, 13] as functional recovery is one of the most important determinants of clinical success. Functional recovery of transplanted allografts depends upon the quality and rate of sensory and motor neural regeneration. The preferred method for neural repair in VCA is primary end-to-end repair, but results using this technique remain suboptimal [133].

ECM-derived biomaterials have been successfully used to promote constructive tissue remodeling in a variety of anatomic locations and tissues including the esophagus [105, 134, 135], urinary tract [136, 137], and musculotendinous tissues [138, 139], among others. These constructive and functional outcomes have consistently been associated with the presence of neural progenitor cell and the formation of functional neuromuscular units [140, 141]. The regenerative potential of ECM-derived biomaterials is due to, at least in part, their ability to retain a number of bioactive molecules [39] that can modulate the host wound healing response upon implantation and promote innervated, vascularized, functional tissue remodeling [39, 96, 142]. The importance of these bioactive molecules has been recently recognized in neural tissue repair [143], and hence, a number of ECM-based technologies to promote nerve regeneration are currently under development.

For example, ECM-based hydrogels derived from urinary bladder ECM (UBM) have been shown to reduce lesion volume and attenuate trauma-induced myelin disruption in a preclinical model of traumatic brain injury [144]. No differences in cognitive recovery were observed between the UBM- and vehicle-treated groups in this study, but the authors report that UBM treatment resulted in significant neurobehavioral recovery as demonstrated by improvements in vestibulomotor function.

A number of studies have suggested that utilizing ECM-derived biomaterials in anatomically homologous structures might offer site-specific advantages when compared to biomaterials derived from heterologous structures [145–148]. Consequently, biologic scaffolds composed of central nervous system (CNS) ECM have been developed and their properties have been studied* in vitro*. Results from these experiments suggest that CNS ECM provides tissue-specific advantages including the ability to stimulate migration of PC12 cells to a greater degree than non-CNS ECMs [149] and the ability to increase neurite length when compared to other non-CNS ECMs [99]. It should be noted, however, that heterologous tissue sources of ECM have also shown robust remodeling effects [93–95, 105].

Biomaterials derived from decellularized neural tissue have also been used clinically to bridge gaps in sensory, motor, and mixed nerves. This technology is limited to the repair of short gaps in peripheral nerves [150] and its application in VCA remains to be investigated. However, in nonneural tissue applications, nerve regeneration has been observed following implantation of ECM-derived scaffolds, and it has been identified as an early predictor of constructive tissue remodeling [140, 151, 152].

Together, these findings suggest that although the process of biomaterial-mediated tissue repair is inherently different from true tissue regeneration, the use ECM-based technologies in VCA can potentially modulate and positively contribute to the process of neural repair.

### 4.4. Donor Graft Manipulation

The objective of the decellularization process through which ECM-derived biomaterials are manufactured is to thoroughly remove proinflammatory cell-associated antigens while at the same time preserving the ultrastructure and composition of the ECM. Properly decellularized biomaterials can function as cell-guiding templates that contain the adequate three-dimensional architecture and biochemical cues to promote cell infiltration and mediate tissue repair upon implantation [39, 91, 96, 142]. Commercially available options (Table 2) typically exist in single sheet form and are normally used to repair tissue defects and approximate wound edges across large tissue gaps. However, more complex ECM-derived scaffolds such as decellularized myocutaneous flaps and composite tissue allografts are currently being developed. Comminuted forms of ECM are also available [153, 154].

Donor graft manipulation relies heavily upon perfusion decellularization, a technique originally developed for whole organ decellularization [155–159]. Using this technique, a number of composite allografts have been decellularized and characterized including fasciocutaneous flaps [160], tendon-bone composite grafts [161], and musculofascial grafts [162], among others.

In a recent study by Jank et al. [163], a bioartificial graft composed of allogeneic ECM and seeded cells was produced via whole limb decellularization. The authors report preservation of the passive musculoskeletal apparatus, nerve sheets, and an intact vasculature through which subsequent cell seeding can be performed. The authors also note that unlike solid organ transplants, anatomic structures transplanted in VCA are not fully functional at the time of transplantation due to a lack of innervation. Functionalization of the resulting ECM construct requires that nerves must first regrow into the transplanted grafts and in the case of bioartificial grafts preserved nerve sheets may act as guiding templates for penetrating axons as they reinnervate sensory and motor organs within the skin [164]. When properly decellularized, ECM-derived materials have inherent immunomodulatory properties that promote constructive tissue remodeling. Bioartificial composite tissue grafts derived from decellularized tissues may be transplanted with or without a cellular component at an early maturation stage and allowed to regenerate and mature* in vivo* but vascularization, reinnervation and functionalization remain the key obstacles.

### 4.5. Current Challenges Associated with ECM-Based Applications for VCA

A number of important obstacles remain before ECM-based technologies are successfully implemented as adjuvant therapy for VCA and become widespread in the clinical setting. Despite promising results obtained through decades of research in the fields of tissue engineering and regenerative medicine, the potential applications of these technologies in VCA are still hypothetical.

ECM-derived hydrogels have been manufactured from multiple tissue sources and their properties have been extensively characterized [98, 99, 119, 120, 165]. However, studies describing ECM-derived hydrogels as minimally invasive drug delivery vehicles are scarce [123]. Although the advantage of minimally invasive implantation of ECM hydrogels is evident, the ability and degree to which these technologies can carry and deliver pharmacological and cellular agents is less clear. Therefore, progress on the use of ECM-derived hydrogels for drug delivery applications will depend upon a number of milestones, including a description of the pharmacokinetic profiles of these materials, including their ability to carry and release specific pharmacological agents (i.e., hydrophilic, hydrophobic, and small and large molecules), and the effect of the degradation process and tissue integration upon these properties [113]. A phase I clinical study with porcine cardiac ECM hydrogel for treatment of damaged myocardium is currently in progress (Ventrix, Inc. Clinical trial identifier: NCT02305602).

In addition, the immunomodulatory properties of ECM-derived biomaterials have been well described only in the setting of tissue engineering and regenerative medicine. In these areas, ECM-derived biomaterials have been shown to promote a microenvironment that is conducive to constructive tissue remodeling via a complex process involving matricryptic peptide release and modulation of the innate immune system. However, the extent to which ECM-derived biomaterials can modulate the immune system and contribute to the promotion of a favorable microenvironment to prevent allograft rejection remains to be determined [96, 166, 167].

Lastly, the optimal anatomic placement of these materials to facilitate allograft survival has not yet been established. Localized drug delivery applications might merely require the material containing the pharmacologic agent to be placed near the periphery of the transplanted graft [15]. The pharmacologic effects of the therapy would thus be localized to the anatomic vicinity of the allograft and systemic exposure would be avoided. Interestingly, studies suggest that localization to regional lymphatic organs, particularly in the case of stem cell delivery, might be an efficacious strategy [69, 168]. In contrast, ECM-derived biomaterials intended for VCA applications unrelated to drug delivery, as in the case of promotion of constructive tissue remodeling and modulation of the immune system via intrinsic properties of the ECM, might need to be implanted at the interphase between the native tissue and the transplanted graft or other locations, depending on the specific application.

## 5. Discussion

Progress in field of regenerative medicine has resulted in emerging implications for VCA. The overlap between these two fields is still nascent and early independent results show great promise in drug delivery, stem cell delivery, tissue regeneration, immunomodulation, and combination of these approaches. The ECM is secreted by the cells of every tissue in an organ and, as such, constitutes the ideal substrate to promote cell proliferation and growth. Furthermore, the immunomodulatory properties of ECM-derived biomaterials that enable them to promote constructive tissue remodeling present clear advantages over synthetically derived biomaterials in VCA applications. However, unlike synthetically derived biomaterials, ECM-derived materials are subject to natural variability depending on the tissue source and species from which they are derived, their methods of manufacture, and their biomechanical properties (i.e., degradation rate, fiber diameter, pore size) are not as controllable and reliably replicated.

It is evident, however, that controlling the microenvironment within each transplant and adjacent regions will be key to successful engraftment and regeneration. These niche conditions include critical variables such as oxygen concentration, cytokine gradients, pH, nutrients availability, microarchitecture, and based composition, all of which are in a state of dynamic equilibrium in temporal and spatial patterns with the host and graft tissues [167].

Despite recent progress in VCA, clinical failures (*many remain unpublished and unpublicized*) have resulted from existing limitations including but not limited to adverse effects of chronic high dose immunosuppression regimens, chronic rejection or uncontrolled acute rejection due to medication nonadherence (which may be underreported in VCA) or other etiologies, and, importantly, suboptimal nerve regeneration with limited functional outcomes in many patients. A number of strategies are currently under development including novel minimally invasive drug delivery systems, tolerance-inducing protocols, and methods to mitigate ischemic reperfusion injury or reduce the antigenic load of transplanted grafts. ECM-derived biomaterials offer unique opportunities to define and control the microenvironment, promote nerve regeneration, and provide a stable substrate for minimally invasive drug and stem cell delivery systems. Despite these advantages, a single technology is unlikely to be effective in all situations. Instead, each tissue and each pathologic condition will likely require a different strategy to obtain optimal results. The viability and sustainability of VCA as a field will rely heavily on strategies that reliably and reproducibly accomplish not only graft survival but also functional outcomes with minimal need or no reliance on systemic immunosuppression with its specter of long term risks.

## Figures and Tables

**Table 1:** Summary of upper extremity transplantation experience.

Country	Center	Total number of limbs	Number of limbs lost	Mortalities
Australia	Melbourne	1		

Austria	Innsbruck	9		

Belgium	Brussels	1		

China	Six centers	15	7	

France	Lyon	11	1	
	Paris	2	2^*∗*^	1^*∗*^

Germany	Munich	2		

India	Kochi	4		

Iran	Tehran	1		

Italy	Milan	3		
	Monza	2		

Malaysia	Selayang	1		

Mexico	Mexico City	4	2	1

Poland	Wroclaw	7	1	

Spain	Madrid	2		
	Valencia	6		

Turkey	Ankara	2	2^*∗*^	1^*∗*^

Turkey	Antalya	6	2^*∗*^	1^*∗*^

United Kingdom	Leeds	1		

United States	Brigham and Women's Hospital, MA	4	2^*∗*^	
	Emory, GA	3	2	
	University of Pittsburgh/Johns Hopkins University	11	2	
	Massachusetts General Hospital, MA	1		
	University of Louisville, KY	9	2	1
	UCLA	1	1	
	University of Pennsylvania	4		
	Wilford Hall, Medical Center, TX	1		

Totals		**114**	**26**	**5**

^*∗*^Simultaneous hand and other body regions transplantation (face or leg). Table adapted from published and presented updates.

**Table 2:** Commercially available ECM-derived products for surgical applications.

Product	Source tissue	Manufacturer	Postprocessing	Form
AlloDerm	Human skin	LifeCell	Natural	Dry scaffold
Bard Dermal Allograft	Human dermis	Bard	Natural	Dry scaffold
CuffPatch	Porcine SIS	Biomet Sports Medicine	Cross-linked	Hydrated scaffold
Dura-Guard	Bovine pericardium	Synovis Surgical Innovations	Cross-linked	Hydrated scaffold
Durasis	Porcine SIS	SIS Cook	Natural	Dry scaffold
Durepair	Bovine fetal skin	TEI Biosciences	Natural	Dry scaffold
FasLata	Human fascia lata	CR Bard	Natural	Dry scaffold
GraftJacket	Human Skin	Wright Medical Tech	Natural	Dry scaffold
MatriStem	Porcine UBM	ACell	Natural	Dry scaffold
Oasis	Porcine SIS	Cook Biotech/Healthpoint	Natural	Dry scaffold
Pelvicol	Porcine dermis	CR Bard	Cross-linked	Hydrated scaffold
Peri-Guard	Bovine pericardium	Synovis Surgical Innovations	Cross-linked	Dry scaffold
Permacol	Porcine skin	Tissue Science Laboratories	Cross-linked	Hydrated scaffold
PriMatrix	Bovine fetal skin	TEI Biosciences	Natural	Dry scaffold
Restore	Porcine SIS	DePuy	Natural	Dry scaffold
Stratasis	Porcine SIS	Cook Biomedical	Natural	Dry scaffold
SurgiMend	Bovine fetal skin	TEI Biosciences	Natural	Dry scaffold
Surgisis	Porcine SIS	Cook Biomedical	Natural	Dry scaffold
Suspend	Human fascia lata	Mentor	Natural	Dry scaffold
TissueMend	Bovine fetal skin	TEI Biosciences	Natural	Dry scaffold
Vascu-Guard	Bovine pericardium	Synovis Surgical Innovations	Cross-linked	Dry scaffold
VentriGel	Porcine myocardium	Ventrix	Gelation	Hydrogel
Veritas	Bovine pericardium	Synovis Surgical Innovations	Cross-linked	Hydrated scaffold
Xenform	Bovine fetal skin	TEI Biosciences	Natural	Dry scaffold
Zimmer Collagen Patch	Porcine dermis	Tissue Science Laboratories	Cross-linked	Hydrated scaffold

Commercially available ECM-derived materials are manufactured from a variety of tissue sources and species and exist in a variety of formats including hydrogels, scaffolds, and cross-linked scaffolds.

**Table 3:** Reports on clinical use of ECM-derived products.

Clinical application	Report
Breast	Butterfield [92]
Dental	Gholami et al. [103]
Diabetic ulcers	Lecheminant and Field [104]
Gastrointestinal	Badylak et al. [105]
Maxillofacial	Leventhal and Pribitkin [94]
Skeletal muscle	Sicari et al. [95]
Urologic	Alpert et al. [106]
Ventral hernia	Kissane and Itani [93]
Vascular	J. M. Ladowski and J. S. Ladowski [107]
Colorectal	Cintron et al. [108]
Thoracic	Scholl et al. [109]

ECM-derived products are routinely used in various anatomic locations for tissue repair.
